# Emerging roles of interferon-stimulated gene-15 in age-related telomere attrition, the DNA damage response, and cardiovascular disease

**DOI:** 10.3389/fcell.2023.1128594

**Published:** 2023-03-21

**Authors:** María González-Amor, Beatriz Dorado, Vicente Andrés

**Affiliations:** ^1^ CIBER Enfermedades Cardiovasculares (CIBERCV), Madrid, Spain; ^2^ Molecular and Genetic Cardiovascular Pathophysiology Laboratory, Novel Mechanisms of Atherosclerosis Program, Centro Nacional de Investigaciones Cardiovasculares (CNIC), Madrid, Spain

**Keywords:** ISG15, aging, telomere shortening, DNA damage response, hypertension, diabetes, obesity

## Abstract

Population aging and age-related cardiovascular disease (CVD) are becoming increasingly prevalent worldwide, generating a huge medical and socioeconomic burden. The complex regulation of aging and CVD and the interaction between these processes are crucially dependent on cellular stress responses. Interferon-stimulated gene-15 (ISG15) encodes a ubiquitin-like protein expressed in many vertebrate cell types that can be found both free and conjugated to lysine residues of target proteins *via* a post-translational process termed ISGylation. Deconjugation of ISG15 (deISGylation) is catalyzed by the ubiquitin-specific peptidase 18 (USP18). The ISG15 pathway has mostly been studied in the context of viral and bacterial infections and in cancer. This minireview summarizes current knowledge on the role of ISG15 in age-related telomere shortening, genomic instability, and DNA damage accumulation, as well as in hypertension, diabetes, and obesity, major CVD risk factors prevalent in the elderly population.

## 1 Introduction

**FIGURE 1:**
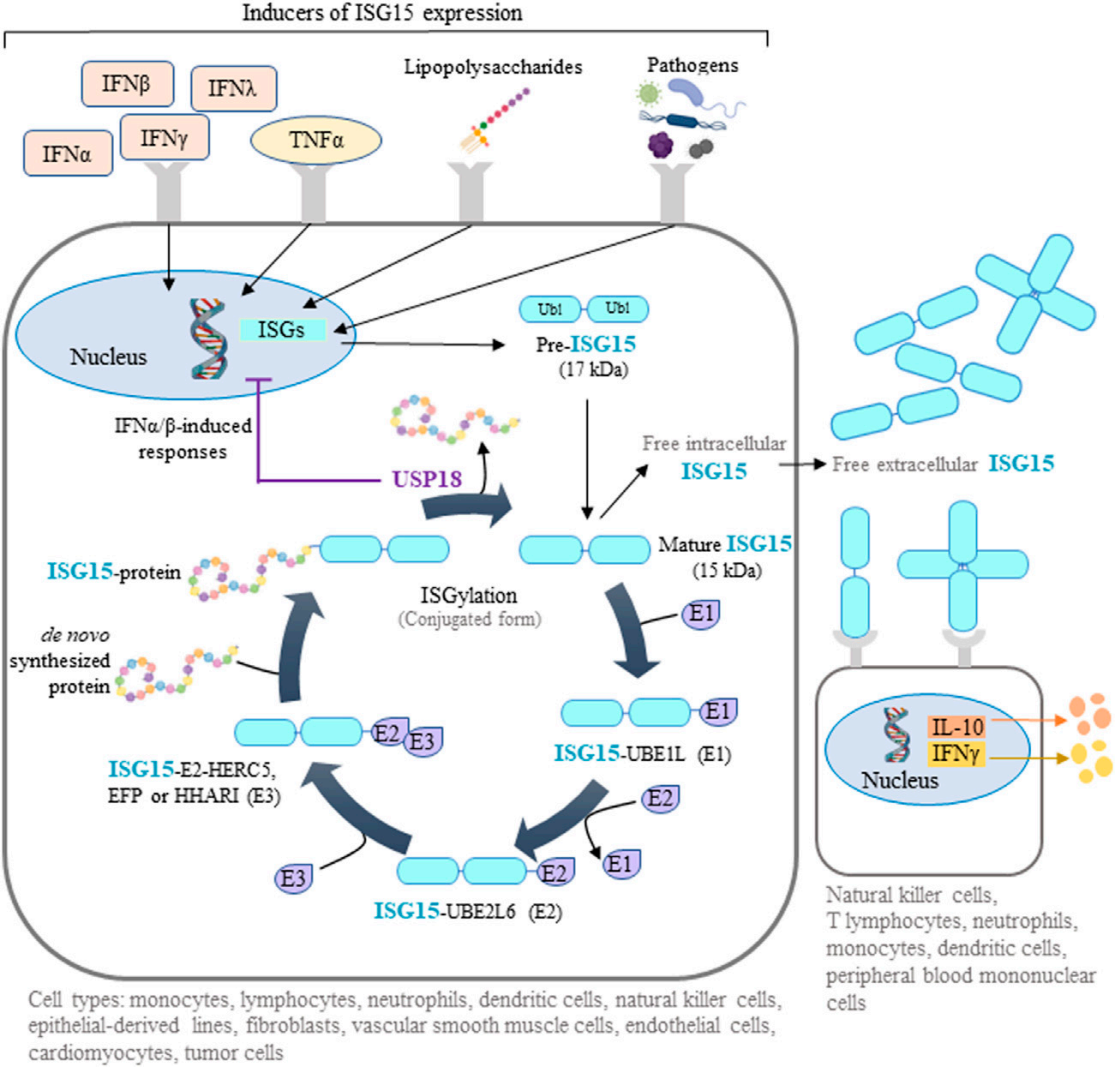
ISG15-mediated protein ISGylation. ISG15 is produced as a 17-kDa precursor with two ubiquitin-like domains in response to diverse stimuli, including interferons (IFNs), tumor necrosis factor *α* (TNFα), lipopolysaccharides, and several pathogens. Intracellular ISG15 exists either as a free protein or conjugated to *de novo* synthesized proteins through ISGylation. This post-translational process can be reversed by the action of ubiquitin specific peptidase 18 (USP18), a protease that also regulates IFN-mediated signaling. Free intracellular ISG15 can be secreted to act as a cytokine, causing the release of IFNγ and interleukin 10 (IL-10). Extracellular ISG15 can form dimers and multimers that modulate cytokine levels. Some illustrations were created using BioRender.com.

Interferon-stimulated gene-15 (*ISG15*) encodes a ubiquitin-like protein expressed in many vertebrate cell types, including monocytes, lymphocytes, neutrophils, dendritic cells, natural killer (NK) cells, epithelial-derived cell lines, fibroblasts, vascular smooth muscle cells, endothelial cells, cardiomyocytes, and some tumor cells (Knight and Cordova, 1991; Zhang and Zhang, 2011; Colonne et al., 2011; Bogunovic et al., 2012; Rahnefeld et al., 2014; Tecalco Cruz and Mejia-Barreto, 2017; Albert et al., 2018; Gonzalez-Amor et al., 2022). Initially produced as a 17 kDa precursor, mature ISG15 is a 15 kDa protein with two ubiquitin-like domains: an N-terminal regulatory domain and a C-terminal conjugating domain (Chang et al., 2008) (Figure 1). ISG15 can be found as a free intracellular or extracellular protein or conjugated to target proteins through a reversible post-translational modification called ISGylation. Similar to ubiquitination, ISGylation of *de novo* synthesized proteins requires ATP-dependent ISG15 conjugation to lysine residues mediated by an enzymatic process involving E1 activating enzyme (UBE1L), E2 conjugating enzyme (UBE2L6), and E3 ligases (HERC5, EFP, HHARI) (Zhang and Zhang, 2011; Durfee and Huibregtse, 2012; Albert et al., 2018). Deconjugation of ISG15 (deISGylation) is catalyzed by the ubiquitin specific peptidase 18 (USP18) (Honke et al., 2016) (Figure 1). ISG15 and the enzymes involved in ISGylation are induced by several interferons (IFNs), mainly type I (IFNα and β) but also type II (IFNγ) and type III (IFNλ), as well as in response to tumor necrosis factor *α* (TNFα), lipopolysaccharides, and several infectious pathogens (Levy et al., 1990; Jeon et al., 2010; Zhang and Zhang, 2011; Chairatvit et al., 2012; MacParland et al., 2016; Albert et al., 2018; Lertsooksawat et al., 2019) (Figure 1). In addition, a feedback loop operates in humans where ISG15 stabilizes USP18, so that intracellular ISG15 deficiency provokes USP18 downregulation (Basters et al., 2018). Remarkably, USP18 can counteract type I IFN-induced responses by a mechanism independent of deISGylation which is mediated by the interaction of USP18 with type I IFN receptor, which in turn inhibits JAK-STAT-dependent signaling (Basters et al., 2018).

Intracellular ISG15 and ISGylation have been extensively studied in the context of viral and bacterial infections. In this context, they generally play a protective role, either directly, by counteracting pathogen activities such as cell entry, nucleic acid trafficking, viral replication and integration, and cell release, or indirectly, by regulating immune-cell IFN production (Kunzi and Pitha, 1996; Yuan and Krug, 2001; Lenschow et al., 2007; Guerra et al., 2008; Hsiang et al., 2009; Villarroya-Beltri et al., 2017). Surprisingly, humans with ISG15 deficiency do not show increased susceptibility to viral infection, possibly due to concomitant reduction of USP18 expression and USP18-dependent negative regulation of IFN-dependent signaling (Speer et al., 2016). Higher expression of ISG15 was found in female than in male HIV-1-infected patients (Chang et al., 2013), however we are unaware of any work reporting gender-dependent differences in ISG15 expression in basal conditions.

Extracellular ISG15 binds to surface receptors such as leukocyte-function associated antigen-1 on various cell types (neutrophils, monocytes, T lymphocytes, NK cells, dendritic cells, and peripheral blood mononuclear cells (PBMCs)), where it triggers the release of interleukins, including IFNγ (Dos Santos and Mansur, 2017; Ostvik et al., 2020) (Figure 1). Interestingly, individuals deficient for secreted, extracellular ISG15 are more susceptible to mycobacterial infection as a result of defective immune responses caused by reduced immune-cell IFN production (Bogunovic et al., 2012).

ISGylation also regulates key cellular processes involved in stress responses, such as proteasomal degradation of misfolded proteins, modulation of autophagy-dependent and lysosome-dependent degradation, downregulation of protein translation, inhibition of exosome secretion, attenuation of hypoxia, and modulation of cytoskeletal dynamics (Villarroya-Beltri et al., 2017; Dzimianski et al., 2019; Sandy et al., 2020) Due to these broad functions, it is unsurprising that ISG15 plays an important role in age-related diseases, including cancer (Zhang and Zhang, 2011), hypertension (Gonzalez-Amor et al., 2022), diabetes (Sun et al., 2019), and obesity (Wei et al., 2021; Yan et al., 2021) (see below).

Aging is a universal, inevitable, and multifactorial degenerative process associated with disability and increased risk of death due to major chronic diseases, including cardiovascular disease (CVD), the leading cause of morbimortality worldwide (Yazdanyar and Newman, 2009; Hamczyk et al., 2018; Galkin et al., 2019). Population aging and CVD prevalence have increased rapidly over the past decades in developed countries and in much of the developing world, creating an enormous medical and socioeconomic burden worldwide. The pace of biological aging (also called functional aging, denoting the decline over time in tissue and organismal function) is influenced by genetic and environmental factors (Hamczyk et al., 2020). Age-related deterioration has been proposed to result from genetic and molecular processes called “hallmarks of aging”, which include genomic instability, telomere attrition, epigenetic alterations, impaired proteostasis, dysregulated nutrient sensing, mitochondrial dysfunction, cell senescence, stem cell exhaustion, and altered intercellular communication (Lopez-Otin et al., 2013). This review discusses studies that link ISG15 to age-related telomere shortening, genomic instability, DNA damage accumulation, and three key CVD risk factors hypertension, diabetes, and obesity.

## 2 ISG15 and age-associated telomere attrition

Preservation of genome stability and integrity in eukaryotes requires functional telomeres. These specialized structures located at both chromosome ends contain multiple non-coding double-stranded repeats of a G-rich DNA sequence that ends in a short single-stranded sequence (Hoffmann et al., 2021). Cells in culture can divide until they reach the Hayflick limit, when they enter in a non-replicative state called senescence/mortality stage 1 (normal cells) or crisis/mortality stage 2 (cells expressing oncogenes) (Hayflick and Moorhead, 1961; Hayflick, 1965; Wei and Sedivy, 1999). Cellular aging and related growth arrest are associated with telomere attrition, chromosomal end-to-end fusions, and apoptosis and senescence when telomere length drops below a critical threshold; however, the order of events and whether telomere ablation is cause or consequence of the age-related alterations remains controversial (Hoffmann et al., 2021).

Telomere attrition can regulate gene expression long before the induction of cell-cycle arrest and DNA damage signaling by critically short telomeres (Robin et al., 2014). Genes in close proximity to telomeres are frequently silenced, a phenomenon known as the “telomere position effect”. In addition, loops formed by long telomeres can interact with and silence genes located as far as 10 Mb from the telomere ends, known as the “telomere position effect over long distances”. Upon telomere attrition, these loops become smaller and cover shorter chromosomal distances. Consequently, genes more distant from telomere ends become released from telomere-loop inhibition and can be expressed (Robin et al., 2014). *ISG15* is located 1 Mb from the end of human chromosome 1p and was the first human gene reported to progressively increase its transcription with the loss of telomere looping upon telomere attrition (Lou et al., 2009; Zhang et al., 2021). This analysis showed that the number of interactions between the *ISG15* locus and the chromosome 1p telomere structure is much higher in young cells than in old ones (Zhang et al., 2021). Free ISG15 expression and ISGylation both increase with telomere shortening in human cells independently of the expression of type I IFNs and of the transcription factor p53 (Lou et al., 2009). Moreover, independently of the telomere position effect, short and/or dysfunctional telomeres can lead to different cellular fates by activating the cyclic GMP-AMP synthase (cGAS)-stimulator of interferon genes (STING) pathway, which engages the autophagy machinery and leads to ISG15 expression and other IFN-stimulated genes (Nassour et al., 2019; Nassour et al., 2023). In patients with COVID-19, the cGAS-STING pathway is also a critical driver of pathological type I IFN responses, which are associated with upregulation of ISG15 and several pro-inflammatory cytokines in skin biopsies (Domizio et al., 2022).

This regulation of *ISG15* expression may therefore promote tumor suppression before additional mechanisms of DNA repair are triggered, and impaired *ISG15* upregulation due to telomere ablation could contribute to the initiation and progression of age-associated diseases (Lou et al., 2009; Robin et al., 2014).

## 3 ISG15 in the age-associated DNA damage response (DDR): p53 and PTEN

During both physiological and premature aging, the accumulation of senescent cells and the onset of age-related disorders are driven by genomic instability provoked by the cumulative action of endogenous and environmental factors and a defective DDR (Liu et al., 2005; Varela et al., 2005; Schumacher et al., 2008; Hoeijmakers, 2009; Cheedipudi et al., 2019; Chen et al., 2019; Wei and Ji, 2018; Di Micco et al., 2021; Schumacher et al., 2021; von Zglinicki et al., 2021). The role of ISG15 and ISGylation in the DDR has mostly been studied in the context of cancer. The ISG15/ISGylation system can be oncogenic or have tumor suppressor activity depending on the tissue affected, cancer stage, and specific cancer-related alterations in signalling pathways (Han et al., 2018; Kang et al., 2022). This complexity might be related to the cross-regulation between ISG15 and key tumor suppressors. One of the few identified and validated ISG15 substrates is the tumor suppressor p53, a pivotal transcription factor that coordinates the expression of many DDR effector genes that induce cell-cycle arrest, DNA repair, autophagy, senescence, and apoptosis (Sandy et al., 2020; Luo et al., 2022). ISG15 and p53 modulate each other at several levels. ISG15 and the ISGylation enzymes UBE1L (E1), UBE2L6 (E2), and EFP (E3) each have a p53-responsive element in their promoters, so their expression increases upon p53 activation (Park et al., 2016). Moreover, DNA damage induces the ISGylation of p53, increasing the transcription of target genes such as *CDK1*, *BAX*, and *MDM2,* as well as that of *ISG15* and ISGylation factors (Park et al., 2016). ISGylation increases p53 activity and reduces the inhibitory activity of ΔNp63α (an alternative splice variant of the p53 family protein p63 that suppresses the trans-activity of other p53 family members), thus promoting cell growth arrest and tumor suppression (Jeon et al., 2012; Park et al., 2016). However, misfolded p53 can undergo proteasomal degradation upon ISGylation mediated by the ISG15 E3 ubiquitin ligase HERC5, and ISG15 deletion in normal cells causes accumulation of misfolded p53 and inhibits p53 activity (Huang et al., 2014). ISG15 and ISGylation therefore have potential as therapeutic targets for the fine-tuning of the p53-dependent DDR.

Another ISG15 substrate in the DDR pathway is the tumor suppressor phosphatase and tensin homolog (PTEN), which is lost in many cancer types (Lee et al., 2018; Alvarez-Garcia et al., 2019) and also plays important roles in diabetes (Li et al., 2017) and autism (Cummings et al., 2022). ISGylation reduces the cytoplasmic content of PTEN, while USP18-mediated deISGylation promotes PTEN protein stability and recovery of its cytoplasmic expression (Mustachio et al., 2017).

In summary, further studies are warranted to determine the role of ISG15/ISGylation in different cancer types, since its effect might be tumor suppressive or oncogenic depending on the context. Moreover, it is important to address the possible role of ISG15 pathway in the premature aging side effects triggered by anti-cancer therapies due to activation of DNA damage responses in healthy tissues.

## 4 ISG15 in age-related cardiovascular and cardiometabolic diseases

Most evidence for a role of ISG15 in CVD is related to viral infection. ISG15 activation is associated with coxsackievirus B3-induced myocarditis in mice (Maier et al., 2012), and cardiomyocyte ISG15 expression contributes significantly to the suppression of viral replication (Rahnefeld et al., 2014). Moreover, ISG15 suppresses viral infection in human cardiomyocytes, and patients with viral cardiomyopathy show conjugated ISG15 induction in the myocardium (Rahnefeld et al., 2014). These findings suggest that ISG15 activation in cardiomyocytes plays an important role in the fight against infectious pathogens, thereby diminishing inflammatory cardiomyopathy, heart failure, and mortality (Rahnefeld et al., 2014). However, the expression of ISG15 pathway components and several inflammatory cytokines is upregulated by cardiomyocyte-specific IkB kinase/NFκB activation, leading to cardiomyopathy and heart failure (Maier et al., 2012). Viruses and viral products can either activate or inhibit the NFκB cascade by direct or indirect binding to IKK subunits to enhance viral replication, evade the innate immune system, and establish an infection (Amaya et al., 2014). In a myocarditis model, cardiac innate immunity is partly mediated by lncRNA metastasis-associated lung adenocarcinoma transcript 1 (MALAT1) (Gast et al., 2016). In line with this finding, an analysis of apolipoprotein E-null mice with heterozygous MALAT1 deficiency revealed massive immune-system dysregulation and exaggerated atherosclerosis even when the mice were kept on a normal diet, and these features were associated with upregulated IFN signaling and *Isg15* expression in splenocytes (Gast et al., 2019). Collectively, these results suggest that ISG15 expression can be both protective and deleterious for the cardiovascular system. In the following sections, we discuss studies that highlight an emerging role of the ISG15 pathway in hypertension, cardiac hypertrophy, diabetes, and obesity.

### 4.1 Hypertension and cardiac hypertrophy

Hypertension is a major risk factor for CVD, kidney disease, stroke, and diabetes (Fuchs and Whelton, 2020; Kim and Kim, 2022). Aging is associated with both hypertension and ISG15 activation. Although telomere length shows high interindividual variability, several population studies have shown an association between telomere ablation and hypertension (Fuster et al., 2007; Liu et al., 2019). For example, Demissie et al. demonstrated an association between hypertension and shorter leukocyte telomeres in men that appeared to be largely due to insulin resistance (Demissie et al., 2006), a disorder frequently associated with hypertension and age-associated diabetes (Saad et al., 2004; Sowers and Frohlich, 2004). Moreover, age-associated oxidative stress and chronic inflammation both contribute to telomere attrition and hypertension (Liu et al., 2019; Gavia-Garcia et al., 2021). However, these studies do not address whether telomere shortening is a cause or a consequence of hypertension.

A recent study of the ISG15 pathway in vascular pathophysiology found that *Isg15* expression is increased in the aortas of hypertensive animals and in angiotensin II-treated vascular cells and macrophages (Gonzalez-Amor et al., 2022). The same study found that *ISG15* expression in human peripheral blood mononuclear cells positively correlated with systolic and diastolic blood pressure and with carotid intima-media thickness and also found elevated *ISG15* expression in human and mouse abdominal aortic aneurysms (Gonzalez-Amor et al., 2022). Moreover, *Isg15-*null mice were found to be protected against angiotensin II-induced hypertension, elastin remodeling, vascular stiffness, endothelial dysfunction, and expression of inflammatory and oxidative stress markers (Gonzalez-Amor et al., 2022). Conversely, excessive ISGylation in USP18^C61A^ mice was associated with enhanced angiotensin II-induced hypertension, vascular fibrosis, inflammation, generation of reactive oxygen species (ROS), elastin breaks, and aortic dilation and rupture (Gonzalez-Amor et al., 2022). Importantly, treatment of angiotensin II-infused USP18^C61A^ mice with the antioxidant tempol improved survival and reduced aneurysm formation and vascular remodeling (Gonzalez-Amor et al., 2022).

Hypertension is frequently associated with cardiac dilation and hypertrophy, and USP18 expression was found to be high both in dilated human hearts and in hypertrophic mice (Ying et al., 2016). USP18-null mice subjected to hypertrophic stimuli underwent exacerbated cardiac remodeling, and, conversely, cardiomyocyte-specific USP18 overexpression abrogated cardiac remodeling and dysfunction under the same pathological stimuli, a protective effect that was associated with inhibition of the transforming growth factor-β–activated kinase 1-p38/c-Jun N-terminal kinase 1/2 signaling cascade (Ying et al., 2016).

### 4.2 Type 2 diabetes mellitus (T2DM) and obesity

T2DM is considered a conventional risk factor for CVD and is associated with aging, obesity, hypertension, and hypercholesterolemia (Martin-Timon et al., 2014; Strain and Paldanius, 2018). Human studies have shown a positive correlation between plasma glucose, obesity, and *Isg15* mRNA levels in peripheral blood mononuclear cells (Gonzalez-Amor et al., 2022). Moreover, mice fed a high-fat diet (HFD) show increased aortic mRNA expression of *Isg15* and other inflammatory genes such as *Ccl2*, *Il1b*, and *Tnfa* (Ballesteros-Martinez et al., 2022)*.* Using GEO data base query and related online analytical tools, Sun et al. identified ISG15 amongst five genes that were related to both T2M and ovarian cancer, and found that ISG15 expression in ovarian cancer biopsies significantly correlated with patient’s survival time (Sun et al., 2019). The authors concluded that genes and proteins involved in glycometabolism are related to ovarian cancer and may serve as potential therapeutic targets. In another study, the innate immune transcription factor IRF3 was shown to strongly repress thermogenic gene expression and oxygen consumption in adipocytes by upregulating ISG15, which ISGylates several glycolytic enzymes, thus decreasing their function and reducing lactate production (Yan et al., 2021). Moreover, *Isg15*-null mice had elevated energy expenditure and developed resistance to diet-induced obesity, which was attributed to lack of ISGylation of glycolytic enzymes in adipocytes (Yan et al., 2021).

A recent study demonstrated elevated expression of the ubiquitin/ISG15-conjugating enzyme UBE2L6 in white adipose tissue from obese mice and humans; moreover, HFD-fed mice with adipose tissue-specific *Ube2l6* ablation had a reduced content of subcutaneous and visceral white adipose tissue, as well as attenuated glucose intolerance, insulin resistance, compensatory hyperinsulinemia, hypercholesterolemia, and hepatic steatosis (Wei et al., 2021). These effects of adipocyte-specific *Ube2l6* were associated with the stabilization of adipose triglyceride lipase (*Atgl*) (Wei et al., 2021).

## 5 Discussion

**FIGURE 2:**
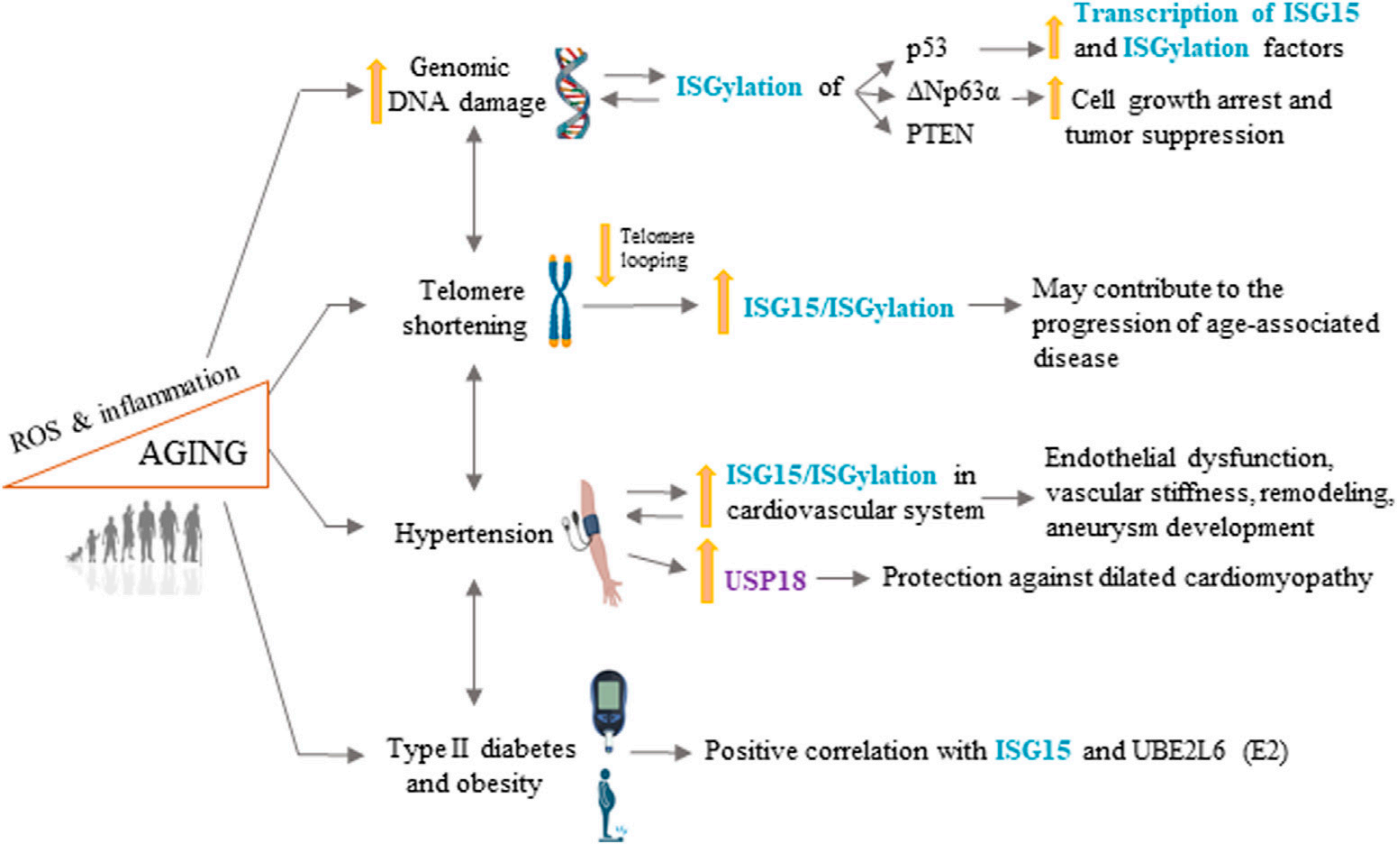
ISG15 in longevity and age-related cardiovascular disease. ISG15 is associated with several processes involved in aging and age-related cardiovascular disease, including increased genomic DNA damage, telomere shortening, hypertension, type II diabetes, and obesity. ROS, reactive oxygen species. Some illustrations were created using BioRender.com.

The world population is aging rapidly, and age is the main risk factor for CVD. Consequently, CVD is the main cause of death in developed societies, where it presents a huge medical and socioeconomic burden. It is therefore of unparalleled importance to understand the cellular and molecular mechanisms governing aging and age-associated CVD. In this review, we have discussed studies that highlight a role of ISG15 as a key element in aging and age-associated CVD beyond its classical roles in the defense against pathogens and as a tumor suppressor. Accumulating evidence links ISG15 to well-characterized aging processes that play major roles in cellular stress responses, such DNA damage, defective DDR, telomere attrition, ROS production, and inflammation, as well as some CVD risk factors (Figure 2). Although targeting ISG15, USP18, and UBE2L6 has the potential to alleviate hypertension, T2DM, and age-associated cardiomyopathies, we are still a long way from understanding the specific consequences of ISGylation on validated substrates (Zhao et al., 2005) and how ISGylation is regulated in a context-specific manner, knowledge that will be essential for the development of ISGylation-targeted therapies.

ISG15 is induced by IFNs and TNFα and can therefore be considered a pro-inflammatory gene. Chronic inflammation and accompanying oxidative stress are thought to contribute to different forms of age-associated CVD (Kofler et al., 2005; Vicenova et al., 2009; Karbach et al., 2014). However, the use of anti-inflammatory drugs or antioxidants to ameliorate CVD has yet to yield conclusive results (Mangge et al., 2014; Kosmas et al., 2019; Mirmiran et al., 2022). For example, treatment with anti-TNFα antibody (infliximab, ATTACH trial) or a soluble TNF receptor (etanercept, RECOVER and RENAISSANCE trials) has shown no effect or even deleterious effects in patients with heart failure (Coletta et al., 2002; Chung et al., 2003). In contrast, treatment of myocardial infarction patients with anti-interleukin-1β antibody (Canakinumab, CANTOS) produced a significant reduction in cardiovascular event rates without changing lipid levels, blood pressure, or incident hypertension (Ridker et al., 2017; Rothman et al., 2020). Future studies should explore the potential benefit of therapies targeting ISG15 and other ISGylation factors as a way to break the vicious cycle of ROS production and inflammation that leads to CVD. However, it may prove difficult to avoid undesired collateral effects of targeting ISG15. Several layers of difficulty are likely to impinge on any therapy targeting ISG15, given its multiple substrates and cellular functions (including pathogen defense and tumor suppression), its context-specific effects, and the complexity of the pro-inflammatory pathways in which it participates. Despite these challenges, further research on ISG15 regulation and functions are likely to shed light on the mechanisms that drive aging and age-associated CVD.

## References

[B1] Albert M. Becares M. Falqui M. Fernandez-Lozano C. Guerra S. (2018). ISG15, a small molecule with huge implications: Regulation of mitochondrial homeostasis. Viruses 10 (11), 629. 10.3390/v10110629 30428561 PMC6265978

[B2] Alvarez-Garcia V. Tawil Y. Wise H. M. Leslie N. R. (2019). Mechanisms of PTEN loss in cancer: It's all about diversity. Semin. Cancer Biol. 59, 66–79. 10.1016/j.semcancer.2019.02.001 30738865

[B3] Amaya M. Keck F. Bailey C. Narayanan A. (2014). The role of the IKK complex in viral infections. Pathog. Dis. 72 (1), 32–44. 10.1111/2049-632X.12210 25082354 PMC7108545

[B4] Ballesteros-Martinez C. Rodrigues-Diez R. Beltran L. M. Moreno-Carriles R. Martinez-Martinez E. Gonzalez-Amor M. (2022). Microsomal prostaglandin E synthase-1 is involved in the metabolic and cardiovascular alterations associated with obesity. Br. J. Pharmacol. 179 (11), 2733–2753. 10.1111/bph.15776 34877656

[B5] Basters A. Knobeloch K. P. Fritz G. (2018). USP18 - a multifunctional component in the interferon response. Biosci. Rep. 38 (6), 1. 10.1042/BSR20180250 PMC6240716 30126853

[B6] Bogunovic D. Byun M. Durfee L. A. Abhyankar A. Sanal O. Mansouri D. (2012). Mycobacterial disease and impaired IFN-gamma immunity in humans with inherited ISG15 deficiency. Science 337 (6102), 1684–1688. 10.1126/science.1224026 22859821 PMC3507439

[B7] Chairatvit K. Wongnoppavich A. Choonate S. (2012). Up-regulation of interferon-stimulated gene15 and its conjugates by tumor necrosis factor-alpha via type I interferon-dependent and -independent pathways. Mol. Cell Biochem. 368 (1-2), 195–201. 10.1007/s11010-012-1360-5 22729740

[B8] Chang J. J. Woods M. Lindsay R. J. Doyle E. H. Griesbeck M. Chan E. S. (2013). Higher expression of several interferon-stimulated genes in HIV-1-infected females after adjusting for the level of viral replication. J. Infect. Dis. 208 (5), 830–838. 10.1093/infdis/jit262 23757341 PMC3733517

[B9] Chang Y. G. Yan X. Z. Xie Y. Y. Gao X. C. Song A. X. Zhang D. E.(2008). Different roles for two ubiquitin-like domains of ISG15 in protein modification. J. Biol. Chem. 283 (19), 13370–13377. 10.1074/jbc.M800162200 18356159

[B10] Cheedipudi S. M. Matkovich S. J. Coarfa C. Hu X. Robertson M. J. Sweet M. (2019). Genomic reorganization of lamin-associated domains in cardiac myocytes is associated with differential gene expression and DNA methylation in human dilated cardiomyopathy. Circ. Res. 124 (8), 1198–1213. 10.1161/CIRCRESAHA.118.314177 30739589 PMC6459729

[B11] Chen S. N. Lombardi R. Karmouch J. Tsai J. Y. Czernuszewicz G. Taylor M. R. G. (2019). DNA damage response/TP53 pathway is activated and contributes to the pathogenesis of dilated cardiomyopathy associated with LMNA (lamin A/C) mutations. Circ. Res. 124 (6), 856–873. 10.1161/CIRCRESAHA.118.314238 30696354 PMC6460911

[B12] Chung E. S. Packer M. Lo K. H. Fasanmade A. A. Willerson J. T. (2003). Anti-TNF therapy against congestive heart failure InvestigatorsRandomized, double-blind, placebo-controlled, pilot trial of infliximab, a chimeric monoclonal antibody to tumor necrosis factor-alpha, in patients with moderate-to-severe heart failure: Results of the anti-TNF therapy against congestive heart failure (ATTACH) trial. Circulation 107 (25), 3133–3140. 10.1161/01.CIR.0000077913.60364.D2 12796126

[B13] Coletta A. P. Clark A. L. Banarjee P. Cleland J. G. (2002). Clinical trials update: RENEWAL (RENAISSANCE and RECOVER) and ATTACH. Eur. J. Heart Fail 4 (4), 559–561. 10.1016/s1388-9842(02)00121-6 12167397

[B14] Colonne P. M. Sahni A. Sahni S. K. (2011). Rickettsia conorii infection stimulates the expression of ISG15 and ISG15 protease UBP43 in human microvascular endothelial cells. Biochem. Biophys. Res. Commun. 416 (1-2), 153–158. 10.1016/j.bbrc.2011.11.015 22100648 PMC3334299

[B15] Cummings K. Watkins A. Jones C. Dias R. Welham A. (2022). Behavioural and psychological features of PTEN mutations: A systematic review of the literature and meta-analysis of the prevalence of autism spectrum disorder characteristics. J. Neurodev. Disord. 14 (1), 1. 10.1186/s11689-021-09406-w 34983360 PMC8903687

[B16] Demissie S. Levy D. Benjamin E. J. Cupples L. A. Gardner J. P. Herbert A. (2006). Insulin resistance, oxidative stress, hypertension, and leukocyte telomere length in men from the Framingham Heart Study. Aging Cell 5 (4), 325–330. 10.1111/j.1474-9726.2006.00224.x 16913878

[B17] Di Micco R. Krizhanovsky V. Baker D. d'Adda di Fagagna F. (2021). Cellular senescence in ageing: From mechanisms to therapeutic opportunities. Nat. Rev. Mol. Cell Biol. 22 (2), 75–95. 10.1038/s41580-020-00314-w 33328614 PMC8344376

[B18] Domizio J. D. Gulen M. F. Saidoune F. Thacker V. V. Yatim A. Sharma K. (2022). The cGAS-STING pathway drives type I IFN immunopathology in COVID-19. Nature 603 (7899), 145–151. 10.1038/s41586-022-04421-w 35045565 PMC8891013

[B19] Dos Santos P. F. Mansur D. S. (2017). Beyond ISGlylation: Functions of free intracellular and extracellular ISG15. J. Interferon Cytokine Res. 37 (6), 246–253. 10.1089/jir.2016.0103 28467275

[B20] Durfee L. A. Huibregtse J. M. (2012). The ISG15 conjugation system. Methods Mol. Biol. 832, 141–149. 10.1007/978-1-61779-474-2_9 22350882 PMC5912894

[B21] Dzimianski J. V. Scholte F. E. M. Bergeron E. Pegan S. D. (2019). ISG15: It's Complicated. J. Mol. Biol. 431 (21), 4203–4216. 10.1016/j.jmb.2019.03.013 30890331 PMC6746611

[B22] Fuchs F. D. Whelton P. K. (2020). High blood pressure and cardiovascular disease. Hypertension 75 (2), 285–292. 10.1161/HYPERTENSIONAHA.119.14240 31865786 PMC10243231

[B23] Fuster J. J. Diez J. Andres V. (2007). Telomere dysfunction in hypertension. J. Hypertens. 25 (11), 2185–2192. 10.1097/HJH.0b013e3282ef6196 17921808

[B24] Galkin F. Zhang B. Dmitriev S. E. Gladyshev V. N. (2019). Reversibility of irreversible aging. Ageing Res. Rev. 49, 104–114. 10.1016/j.arr.2018.11.008 30513346

[B25] Gast M. Rauch B. H. Nakagawa S. Haghikia A. Jasina A. Haas J. (2019). Immune system-mediated atherosclerosis caused by deficiency of long non-coding RNA MALAT1 in ApoE-/-mice. Cardiovasc Res. 115 (2), 302–314. 10.1093/cvr/cvy202 30101304

[B26] Gast M. Schroen B. Voigt A. Haas J. Kuehl U. Lassner D. (2016). Long noncoding RNA MALAT1-derived mascRNA is involved in cardiovascular innate immunity. J. Mol. Cell Biol. 8 (2), 178–181. 10.1093/jmcb/mjw003 26823496 PMC6283121

[B27] Gavia-Garcia G. Rosado-Perez J. Arista-Ugalde T. L. Aguiniga-Sanchez I. Santiago-Osorio E. Mendoza-Nunez V. M. (2021). Telomere length and oxidative stress and its relation with metabolic syndrome components in the aging. Biol. (Basel). 10 (4), 253. 10.3390/biology10040253 PMC8063797 33804844

[B28] Gonzalez-Amor M. Garcia-Redondo A. B. Jorge I. Zalba G. Becares M. Ruiz-Rodriguez M. J. (2022). Interferon-stimulated gene 15 pathway is a novel mediator of endothelial dysfunction and aneurysms development in angiotensin II infused mice through increased oxidative stress. Cardiovasc Res. 118 (16), 3250–3268. 10.1093/cvr/cvab321 34672341 PMC9799052

[B29] Guerra S. Caceres A. Knobeloch K. P. Horak I. Esteban M. (2008). Vaccinia virus E3 protein prevents the antiviral action of ISG15. PLoS Pathog. 4 (7), e1000096. 10.1371/journal.ppat.1000096 18604270 PMC2434199

[B30] Hamczyk M. R. del Campo L. Andres V. (2018). Aging in the cardiovascular system: Lessons from hutchinson-gilford Progeria syndrome. Annu. Rev. Physiol. 80, 27–48. 10.1146/annurev-physiol-021317-121454 28934587

[B31] Hamczyk M. R. Nevado R. M. Barettino A. Fuster V. Andres V. (2020). Biological versus chronological aging: JACC focus seminar. J. Am. Coll. Cardiol. 75 (8), 919–930. 10.1016/j.jacc.2019.11.062 32130928

[B32] Han H. G. Moon H. W. Jeon Y. J. (2018). ISG15 in cancer: Beyond ubiquitin-like protein. Cancer Lett. 438, 52–62. 10.1016/j.canlet.2018.09.007 30213559

[B33] Hayflick L. Moorhead P. S. (1961). The serial cultivation of human diploid cell strains. Exp. Cell Res. 25, 585–621. 10.1016/0014-4827(61)90192-6 13905658

[B34] Hayflick L. (1965). The limited *in vitro* lifetime of human diploid cell strains. Exp. Cell Res. 37, 614–636. 10.1016/0014-4827(65)90211-9 14315085

[B35] Hoeijmakers J. H. (2009). DNA damage, aging, and cancer. N. Engl. J. Med. 361 (15), 1475–1485. 10.1056/NEJMra0804615 19812404

[B36] Hoffmann J. Richardson G. Haendeler J. Altschmied J. Andres V. Spyridopoulos I. (2021). Telomerase as a therapeutic target in cardiovascular disease. Arterioscler. Thromb. Vasc. Biol. 41 (3), 1047–1061. 10.1161/ATVBAHA.120.315695 33504179

[B37] Honke N. Shaabani N. Zhang D. E. Hardt C. Lang K. S. (2016). Multiple functions of USP18. Cell Death Dis. 7 (11), e2444. 10.1038/cddis.2016.326 27809302 PMC5260889

[B38] Hsiang T. Y. Zhao C. Krug R. M. (2009). Interferon-induced ISG15 conjugation inhibits influenza A virus gene expression and replication in human cells. J. Virol. 83 (12), 5971–5977. 10.1128/JVI.01667-08 19357168 PMC2687383

[B39] Huang Y. F. Wee S. Gunaratne J. Lane D. P. Bulavin D. V. (2014). Isg15 controls p53 stability and functions. Cell Cycle 13 (14), 2200–2210. 10.4161/cc.29209 24844324 PMC4111675

[B40] Jeon Y. J. Jo M. G. Yoo H. M. Hong S. H. Park J. M. Ka S. H. (2012). Chemosensitivity is controlled by p63 modification with ubiquitin-like protein ISG15. J. Clin. Invest. 122 (7), 2622–2636. 10.1172/JCI61762 22706304 PMC3386819

[B41] Jeon Y. J. Yoo H. M. Chung C. H. (2010). ISG15 and immune diseases. Biochim. Biophys. Acta 1802 (5), 485–496. 10.1016/j.bbadis.2010.02.006 20153823 PMC7127291

[B42] Kang J. A. Kim Y. J. Jeon Y. J. (2022). The diverse repertoire of ISG15: More intricate than initially thought. Exp. Mol. Med. 54 (11), 1779–1792. 10.1038/s12276-022-00872-3 36319753 PMC9722776

[B43] Karbach S. Wenzel P. Waisman A. Munzel T. Daiber A. (2014). eNOS uncoupling in cardiovascular diseases--the role of oxidative stress and inflammation. Curr. Pharm. Des. 20 (22), 3579–3594. 10.2174/13816128113196660748 24180381

[B44] Kim H. J. Kim K. I. (2022). Blood pressure target in type 2 diabetes mellitus. Diabetes Metab. J. 46 (5), 667–674. 10.4093/dmj.2022.0215 36193727 PMC9532171

[B45] Knight E. Jr. Cordova B. (1991). IFN-induced 15-kDa protein is released from human lymphocytes and monocytes. J. Immunol. 146 (7), 2280–2284. 10.4049/jimmunol.146.7.2280 2005397

[B46] Kofler S. Nickel T. Weis M. (2005). Role of cytokines in cardiovascular diseases: A focus on endothelial responses to inflammation. Clin. Sci. (Lond). 108 (3), 205–213. 10.1042/CS20040174 15540988

[B47] Kosmas C. E. Silverio D. Sourlas A. Montan P. D. Guzman E. Garcia M. J. (2019). Anti-inflammatory therapy for cardiovascular disease. Ann. Transl. Med. 7 (7), 147. 10.21037/atm.2019.02.34 31157268 PMC6511577

[B48] Kunzi M. S. Pitha P. M. (1996). Role of interferon-stimulated gene ISG-15 in the interferon-omega-mediated inhibition of human immunodeficiency virus replication. J. Interferon Cytokine Res. 16 (11), 919–927. 10.1089/jir.1996.16.919 8938567

[B49] Lee Y. R. Chen M. Pandolfi P. P. (2018). The functions and regulation of the PTEN tumour suppressor: New modes and prospects. Nat. Rev. Mol. Cell Biol. 19 (9), 547–562. 10.1038/s41580-018-0015-0 29858604

[B50] Lenschow D. J. Lai C. Frias-Staheli N. Giannakopoulos N. V. Lutz A. Wolff T. (2007). IFN-stimulated gene 15 functions as a critical antiviral molecule against influenza, herpes, and Sindbis viruses. Proc. Natl. Acad. Sci. U. S. A. 104 (4), 1371–1376. 10.1073/pnas.0607038104 17227866 PMC1783119

[B51] Lertsooksawat W. Wongnoppavich A. Chairatvit K. (2019). Up-regulation of interferon-stimulated gene 15 and its conjugation machinery, UbE1L and UbcH8 expression by tumor necrosis factor-alpha through p38 MAPK and JNK signaling pathways in human lung carcinoma. Mol. Cell Biochem. 462 (1-2), 51–59. 10.1007/s11010-019-03609-5 31428903

[B52] Levy D. E. Lew D. J. Decker T. Kessler D. S. Darnell J. E. (1990). Synergistic interaction between interferon-alpha and interferon-gamma through induced synthesis of one subunit of the transcription factor ISGF3. EMBO J. 9 (4), 1105–1111. 10.1002/j.1460-2075.1990.tb08216.x 2108862 PMC551785

[B53] Li A. Qiu M. Zhou H. Wang T. Guo W. P. T. E. N. (2017). PTEN, insulin resistance and cancer. Curr. Pharm. Des. 23 (25), 3667–3676. 10.2174/1381612823666170704124611 28677502

[B54] Liu B. Wang J. Chan K. M. Tjia W. M. Deng W. Guan X. (2005). Genomic instability in laminopathy-based premature aging. Nat. Med. 11 (7), 780–785. 10.1038/nm1266 15980864

[B55] Liu P. Zhang Y. Ma L. (2019). Telomere length and associated factors in older adults with hypertension. J. Int. Med. Res. 47 (11), 5465–5474. 10.1177/0300060519882570 31662013 PMC6862919

[B56] Lopez-Otin C. Blasco M. A. Partridge L. Serrano M. Kroemer G. (2013). The hallmarks of aging. Cell 153 (6), 1194–1217. 10.1016/j.cell.2013.05.039 23746838 PMC3836174

[B57] Lou Z. Wei J. Riethman H. Baur J. A. Voglauer R. Shay J. W. (2009). Telomere length regulates ISG15 expression in human cells. Aging (Albany NY) 1 (7), 608–621. 10.18632/aging.100066 20157543 PMC2806043

[B58] Luo Q. Sun W. Wang Y. F. Li J. Li D. W. (2022). Association of p53 with neurodegeneration in Parkinson's disease. Park. Dis. 2022, 6600944. 10.1155/2022/6600944 PMC9117072 35601652

[B59] MacParland S. A. Ma X. Z. Chen L. Khattar R. Cherepanov V. Selzner M. (2016). Lipopolysaccharide and tumor necrosis factor alpha inhibit interferon signaling in hepatocytes by increasing ubiquitin-like protease 18 (USP18) expression. J. Virol. 90 (12), 5549–5560. 10.1128/JVI.02557-15 27009955 PMC4886784

[B60] Maier H. J. Schips T. G. Wietelmann A. Kruger M. Brunner C. Sauter M. (2012). Cardiomyocyte-specific IκB kinase (IKK)/NF-κB activation induces reversible inflammatory cardiomyopathy and heart failure. Proc. Natl. Acad. Sci. U. S. A. 109 (29), 11794–11799. 10.1073/pnas.1116584109 22753500 PMC3406816

[B61] Mangge H. Becker K. Fuchs D. Gostner J. M. (2014). Antioxidants, inflammation and cardiovascular disease. World J. Cardiol. 6 (6), 462–477. 10.4330/wjc.v6.i6.462 24976919 PMC4072837

[B62] Martin-Timon I. Sevillano-Collantes C. Segura-Galindo A. Del Canizo-Gomez F. J. (2014). Type 2 diabetes and cardiovascular disease: Have all risk factors the same strength? World J. Diabetes 5 (4), 444–470. 10.4239/wjd.v5.i4.444 25126392 PMC4127581

[B63] Mirmiran P. Hosseini-Esfahani F. Esfandiar Z. Hosseinpour-Niazi S. Azizi F. (2022). Associations between dietary antioxidant intakes and cardiovascular disease. Sci. Rep. 12 (1), 1504. 10.1038/s41598-022-05632-x 35087166 PMC8795399

[B64] Mustachio L. M. Kawakami M. Lu Y. Rodriguez-Canales J. Mino B. Behrens C. (2017). The ISG15-specific protease USP18 regulates stability of PTEN. Oncotarget 8 (1), 3–14. 10.18632/oncotarget.13914 27980214 PMC5352120

[B65] Nassour J. Aguiar L. G. Correia A. Schmidt T. T. Mainz L. Przetocka S. (2023). Telomere-to-mitochondria signalling by ZBP1 mediates replicative crisis. Nature 614 (7949), 767–773. 10.1038/s41586-023-05710-8 36755096 PMC9946831

[B66] Nassour J. Radford R. Correia A. Fuste J. M. Schoell B. Jauch A. (2019). Autophagic cell death restricts chromosomal instability during replicative crisis. Nature 565 (7741), 659–663. 10.1038/s41586-019-0885-0 30675059 PMC6557118

[B67] Ostvik A. E. Svendsen T. D. Granlund A. V. B. Doseth B. Skovdahl H. K. Bakke I. (2020). Intestinal epithelial cells express immunomodulatory ISG15 during active ulcerative colitis and crohn's disease. J. Crohns Colitis 14 (7), 920–934. 10.1093/ecco-jcc/jjaa022 32020185 PMC7392169

[B68] Park J. H. Yang S. W. Park J. M. Ka S. H. Kim J. H. Kong Y. Y. (2016). Positive feedback regulation of p53 transactivity by DNA damage-induced ISG15 modification. Nat. Commun. 7, 12513. 10.1038/ncomms12513 27545325 PMC4996943

[B69] Rahnefeld A. Klingel K. Schuermann A. Diny N. L. Althof N. Lindner A. (2014). Ubiquitin-like protein ISG15 (interferon-stimulated gene of 15 kDa) in host defense against heart failure in a mouse model of virus-induced cardiomyopathy. Circulation 130 (18), 1589–1600. 10.1161/CIRCULATIONAHA.114.009847 25165091

[B70] Ridker P. M. Everett B. M. Thuren T. MacFadyen J. G. Chang W. H. Ballantyne C. (2017). Antiinflammatory therapy with canakinumab for atherosclerotic disease. N. Engl. J. Med. 377 (12), 1119–1131. 10.1056/nejmoa1707914 28845751

[B71] Robin J. D. Ludlow A. T. Batten K. Magdinier F. Stadler G. Wagner K. R. (2014). Telomere position effect: Regulation of gene expression with progressive telomere shortening over long distances. Genes Dev. 28 (22), 2464–2476. 10.1101/gad.251041.114 25403178 PMC4233240

[B72] Rothman A. M. MacFadyen J. Thuren T. Webb A. Harrison D. G. Guzik T. J. (2020). Effects of interleukin-1β inhibition on blood pressure, incident hypertension, and residual inflammatory risk: A secondary analysis of CANTOS. Hypertension 75 (2), 477–482. 10.1161/HYPERTENSIONAHA.119.13642 31884854 PMC7055941

[B73] Saad M. F. Rewers M. Selby J. Howard G. Jinagouda S. Fahmi S. (2004). Insulin resistance and hypertension: The insulin resistance atherosclerosis study. Hypertension 43 (6), 1324–1331. 10.1161/01.HYP.0000128019.19363.f9 15123571

[B74] Sandy Z. da Costa I. C. Schmidt C. K. (2020). More than meets the ISG15: Emerging roles in the DNA damage response and beyond. Biomolecules 10 (11), 1557. 10.3390/biom10111557 33203188 PMC7698331

[B75] Schumacher B. Garinis G. A. Hoeijmakers J. H. (2008). Age to survive: DNA damage and aging. Trends Genet. 24 (2), 77–85. 10.1016/j.tig.2007.11.004 18192065

[B76] Schumacher B. Pothof J. Vijg J. Hoeijmakers J. H. J. (2021). The central role of DNA damage in the ageing process. Nature 592 (7856), 695–703. 10.1038/s41586-021-03307-7 33911272 PMC9844150

[B77] Sowers J. R. Frohlich E. D. (2004). Insulin and insulin resistance: Impact on blood pressure and cardiovascular disease. Med. Clin. North Am. 88 (1), 63–82. 10.1016/s0025-7125(03)00128-7 14871051

[B78] Speer S. D. Li Z. Buta S. Payelle-Brogard B. Qian L. Vigant F. (2016). ISG15 deficiency and increased viral resistance in humans but not mice. Nat. Commun. 7, 11496. 10.1038/ncomms11496 27193971 PMC4873964

[B79] Strain W. D. Paldanius P. M. (2018). Diabetes, cardiovascular disease and the microcirculation. Cardiovasc Diabetol. 17 (1), 57. 10.1186/s12933-018-0703-2 29669543 PMC5905152

[B80] Sun Y. Xiaoyan H. Yun L. Chaoqun L. Jialing W. Liu Y. (2019). Identification of key candidate genes and pathways for relationship between ovarian cancer and diabetes mellitus using bioinformatical analysis. Asian Pac J. Cancer Prev. 20 (1), 145–155. 10.31557/APJCP.2019.20.1.145 30678426 PMC6485580

[B81] Tecalco Cruz A. C. Mejia-Barreto K. (2017). Cell type-dependent regulation of free ISG15 levels and ISGylation. J. Cell Commun. Signal 11 (2), 127–135. 10.1007/s12079-017-0385-7 28285335 PMC5440350

[B82] Varela I. Cadinanos J. Pendas A. M. Gutierrez-Fernandez A. Folgueras A. R. Sanchez L. M. (2005). Accelerated ageing in mice deficient in Zmpste24 protease is linked to p53 signalling activation. Nature 437 (7058), 564–568. 10.1038/nature04019 16079796

[B83] Vicenova B. Vopalensky V. Burysek L. Pospisek M. (2009). Emerging role of interleukin-1 in cardiovascular diseases. Physiol. Res. 58 (4), 481–498. 10.33549/physiolres.931673 19093736

[B84] Villarroya-Beltri C. Guerra S. Sanchez-Madrid F. (2017). ISGylation - a key to lock the cell gates for preventing the spread of threats. J. Cell Sci. 130 (18), 2961–2969. 10.1242/jcs.205468 28842471

[B85] von Zglinicki T. Wan T. Miwa S. (2021). Senescence in post-mitotic cells: A driver of aging? Antioxid. Redox Signal 34 (4), 308–323. 10.1089/ars.2020.8048 32164429 PMC7821432

[B86] Wei W. Ji S. (2018). Cellular senescence: Molecular mechanisms and pathogenicity. J. Cell Physiol. 233 (12), 9121–9135. 10.1002/jcp.26956 30078211

[B87] Wei W. Li Y. Li Y. Li D. (2021). Adipose-specific knockout of ubiquitin-conjugating enzyme E2L6 (Ube2l6) reduces diet-induced obesity, insulin resistance, and hepatic steatosis. J. Pharmacol. Sci. 145 (4), 327–334. 10.1016/j.jphs.2020.12.008 33712284

[B88] Wei W. Sedivy J. M. (1999). Differentiation between senescence (M1) and crisis (M2) in human fibroblast cultures. Exp. Cell Res. 253 (2), 519–522. 10.1006/excr.1999.4665 10585275

[B89] Yan S. Kumari M. Xiao H. Jacobs C. Kochumon S. Jedrychowski M. (2021). IRF3 reduces adipose thermogenesis via ISG15-mediated reprogramming of glycolysis. J. Clin. Invest. 131 (7), e144888. 10.1172/JCI144888 33571167 PMC8011904

[B90] Yazdanyar A. Newman A. B. (2009). The burden of cardiovascular disease in the elderly: Morbidity, mortality, and costs. Clin. Geriatr. Med. 25 (4), 563–577. 10.1016/j.cger.2009.07.007 19944261 PMC2797320

[B91] Ying X. Zhao Y. Yao T. Yuan A. Xu L. Gao L. (2016). Novel protective role for ubiquitin-specific protease 18 in pathological cardiac remodeling. Hypertension 68 (5), 1160–1170. 10.1161/HYPERTENSIONAHA.116.07562 27572150

[B92] Yuan W. Krug R. M. (2001). Influenza B virus NS1 protein inhibits conjugation of the interferon (IFN)-induced ubiquitin-like ISG15 protein. EMBO J. 20 (3), 362–371. 10.1093/emboj/20.3.362 11157743 PMC133459

[B93] Zhang D. Zhang D. E. (2011). Interferon-stimulated gene 15 and the protein ISGylation system. J. Interferon Cytokine Res. 31 (1), 119–130. 10.1089/jir.2010.0110 21190487 PMC3021351

[B94] Zhang N. Li Y. Lai T. P. Shay J. W. Danuser G. (2021). Imaging assay to probe the role of telomere length shortening on telomere-gene interactions in single cells. Chromosoma 130 (1), 61–73. 10.1007/s00412-020-00747-4 33555479 PMC7889534

[B95] Zhao C. Denison C. Huibregtse J. M. Gygi S. Krug R. M. (2005). Human ISG15 conjugation targets both IFN-induced and constitutively expressed proteins functioning in diverse cellular pathways. Proc. Natl. Acad. Sci. U. S. A. 102 (29), 10200–10205. 10.1073/pnas.0504754102 16009940 PMC1177427

